# The construction of oligonucleotide-cycloastragenol and the renoprotective effect study

**DOI:** 10.3389/fbioe.2022.1027517

**Published:** 2022-11-28

**Authors:** Lumin Tang, Xiang Li, Yu Qin, Xinyao Geng, Ruowen Wang, Weihong Tan, Shan Mou

**Affiliations:** ^1^ Molecular Cell Lab for Kidney Disease, Ren Ji Hospital, School of Medicine, Shanghai Jiao Tong University, Shanghai, China; ^2^ Shanghai University of Traditional Chinese Medicine, Shanghai, China; ^3^ Ren Ji Hospital, School of Medicine, Institute of Molecular Medicine, Shanghai Jiao Tong University, Shanghai, China; ^4^ State Key Laboratory of Oncogenes and Related Genes, Ren Ji Hospital, School of Medicine, Institute of Molecular Medicine (IMM), Shanghai Jiao Tong University, Shanghai, China; ^5^ Molecular Science and Biomedicine Laboratory (MBL), State Key Laboratory of Chemo/Biosensing and Chemometrics, College of Chemistry and Chemical Engineering, College of Biology, and Aptamer Engineering Center of Hunan Province, Hunan University, Changsha, China

**Keywords:** oligonucleotide synthesis, cycloastragenol, renoprotective effect, phosphoramidite, traditional Chinese medicine

## Abstract

Traditional Chinese Medicine (TCM) provides unique therapeutic effects for many diseases with identified efficacy during long practice. Astragalus Membranaceus (AM) is the Chinese herbal applied for kidney injury in the clinic, but it remains challenging to further enhance the efficacy. Cycloastragenol (CAG) is the ingredient isolated from AM with poor water solubility, which has shown a renoprotective effect. Herein we designed and synthesized the corresponding solid-phase module of CAG, from which CAG as a pharmaceutical element was incorporated into oligonucleotides (ON) as an ON-CAG conjugate in a programmable way by a DNA synthesizer. Cell viability study demonstrated that ON-CAG conjugate remains similar renoprotective effect as that of CAG, which efficiently recovers the activity of HK-2 cells pretreated with cisplatin. Similarly, in the renal cells treated with the conjugate, the biomarkers of kidney injury such as KIM-1 and IL-18 are downregulated, and cytokines are reduced as treated with anti-inflammatory agents. Overall, we have managed to incorporate a hydrophobic ingredient of TCM into ON and demonstrate the oligonucleotide synthesis technology as a unique approach for the mechanism study of TCM, which may facilitate the discovery of new therapeutics based on TCM.

## Introduction

Acute kidney injury (AKI) is a clinical syndrome characterized by a rapid decline in kidney function within a short period, with high incidence and poor prognosis (Hu et al., 2019). There are many causes of AKI and drug use is one of them. The kidney is a target organ for a variety of chemicals including cisplatin poisoning and side effects. Cisplatin, a widely used anti-cancer chemotherapeutic, is mainly excreted through the kidney, depending on the filtration of the glomerulus or the secretion of renal tubules, which is more nephrotoxic and prone to kidney injury (Miller et al., 2010). At present, there is no effective treatment for AKI caused by cisplatin.

Astragalus Membranaceus (AM), a valuable Chinese herb, has been widely used as herbal medicine to treat various diseases of the kidney, cardiovascular system, and nervous system (Tu et al., 2013; Liu et al., 2016; Xu et al., 2019). It has been reported that AM has antioxidant effects, reduces ischemia and reperfusion injury in the heart and brain, and has protective effects against kidney diseases (Zhang et al., 2006; Qu et al., 2019; Li et al., 2020). The previous research results of our team also demonstrated that AM could inhibit the apoptosis of tubular epithelial cells induced by high glucose and attenuate cisplatin-mediated renal injury (Wang et al., 2014; Yan et al., 2017). Astragaloside IV (AS-IV) is a saponin and serves as the predominant constituent of AM. Since most of the AS-IV is metabolized to cycloastragenol (CAG) *in vivo*, CAG is also defined as a natural active compound in AM and an active form of AS-IV (Zhou et al., 2012; Zhao et al., 2015). However, the CAG powder is extremely difficult to dissolve in water and the reported approximate solubility of CAG powder in water is 1:10000. Its poor water solubility restricts direct clinical applications of CAG, which is an urgent problem to be solved at present.

Oligonucleotide (ON) synthesis is the chemical synthesis of relatively short fragments of nucleic acids with a defined chemical structure (Kosuri and Church, 2014). The technique provides rapid and inexpensive access to customized oligonucleotides of the desired sequence and plays an important role in the treatment of many diseases as gene therapy strategies. AS1411 is a 26-base guanine oligonucleotide with the sequence 5′-GGT​GGT​GGT​GGT​TGT​GGT​GGT​GGT​GG-3′ and binds specifically to the nucleolin protein, which is mainly found in the nucleolus of normal cells but is highly expressed on the surface of kidney tumor cells (Koutsioumpa and Papadimitriou, 2014; Yazdian-Robati et al., 2020). The findings previously reported indicated that fluorescence-labeled AS1411 was mainly taken up by cells through endocytosis (Reyes-Reyes et al., 2010).

Based on reported studies, we designed and synthesized a corresponding solid-phase module of CAG, from which CAG as a pharmaceutical element can be incorporated into AS1411 in a programmable way (Zhang et al., 2017; Wang et al., 2021). In this way, we can increase the solubility of CAG and enhance its bioavailability. This study provides a new approach to developing a reliable, convenient, and cost-effective approach to facilitate the discovery of new therapeutics based on TCM.

## Materials and methods

### Synthesis of cycloastragenol phosphoramidite

**FIGURE 1:**
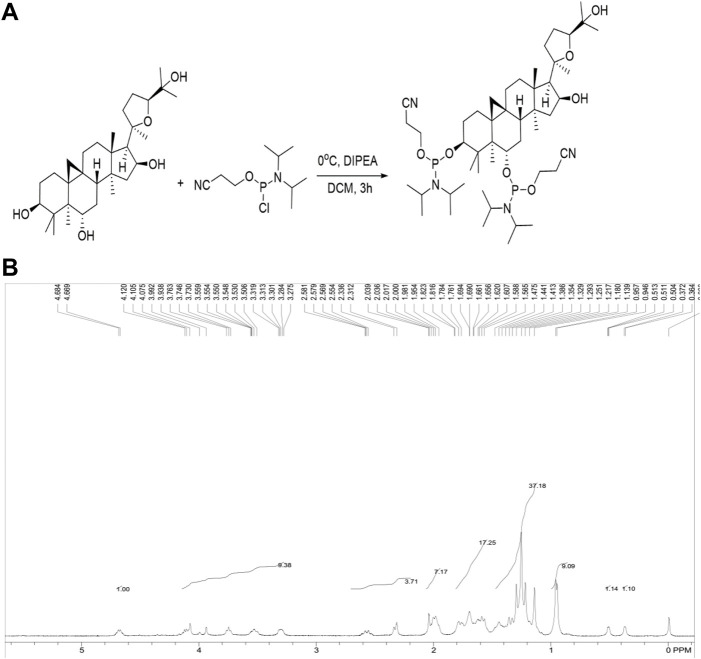
**(A)** Synthesis of CAG phosphoramidite. Under specific condition CAG phosphoramidite was synthesized by combining CAG with 2-cyanoethyl-N, N-diisopropylchlorophosphoramidite. **(B)** ^1^HNMR spectrum of CAG phosphoramidite.

As displayed in Figure 1A, the solution of CAG (491 mg, 1 mmol) in methylene dichloride (5 ml) was placed in a round-bottom flask, purged with N_2_, and cooled to 0°C. After the addition of N,N-diisopropylethylamine (1 ml), 2-cyanoethyl-N,N-diisopro-pylchlorophosphoramidite (521 mg, 2.2 mmol) was added dropwise, and the solution was allowed to warm to room temperature, followed by stirring for 3 h. The mixture was diluted with CH_2_Cl_2_ and washed three times with saturated NaHCO_3_ solution and brine. The combined organic phase was dried over anhydrous Na_2_SO_4_ and concentrated by a rotary evaporator. The residue was purified by flash chromatography (hexanes/EtOAc 9:1 to 6:1 to 3:1 + 1% triethylamine) to give CAG phosphoramidite as a white foam (704 mg, 79%). As determined by _1_H NMR (Figure 1B), CAG phosphoramidite was prepared as a mixture of diastereoisomers with two hydroxyl groups protected. 1H NMR (400 MHz, CDCl3) δ 4.67 (1H), 3.27-4.12 (9H), 2.31-2.58 (4H), 1.98-2.04 (7H), 1.56-1.82 (17H), 1.14-1.47 (37H), 0.95-0.96 (9H), 0.36-0.51 (2H).

### ON-cycloastragenol synthesis and purification DNA preparation

**FIGURE 2:**
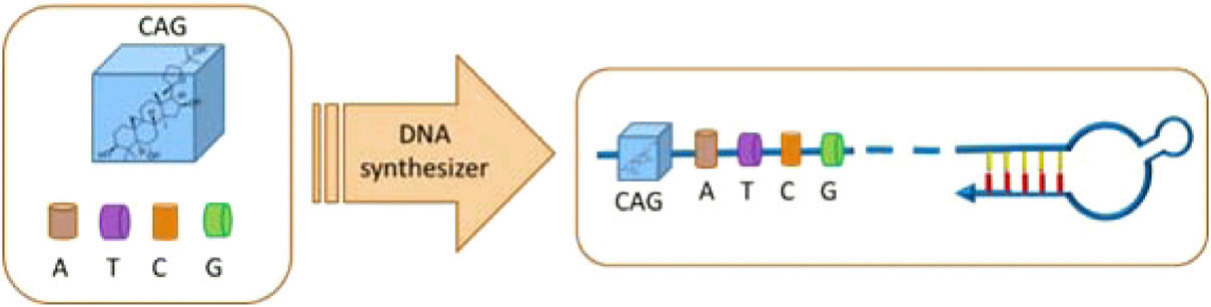
Schematic illustration for the synthesis process of ON-CAG conjugates. CAG phosphoramidite was ligated to the oligonucleic acid sequence by a DNA synthesizer to form ON-CAG conjugates.

Oligonucleotides were prepared with an Applied Biosystems (ABI) 394 DNA/RNA synthesizer. ESI-MS spectra of oligonucleotides were performed by the Shanghai Sangon Mass Spectrometry Facility. All DNA synthesis reagents were purchased from Glen Research. The oligonucleotides were then deprotected in saturated ammonium hydroxide at room temperature for 12 h and further purified by reversed-phase HPLC on a C-18 column using 0.1 M triethylamine acetate (TEAA) buffer and acetonitrile as the eluents. The collected DNA products were dried and detritylated by dissolving and incubating DNA products in 200 μl of 80% acetic acid for 20 min. The detritylated DNA product was precipitated with NaCl (3 M, 25 μl) and ethanol (600 μl) and then desalted on a Glen-Pak DNA Purification Cartridge. The synthetic process of ON-CAG conjugates was displayed in Figure 2.

### Cell culture

HK-2 cells, a human kidney tubular epithelial cell line, were obtained from American Type Culture Collection (ATCC; Manassas, VA, United States) and grown in DMEM/F12 containing 10% FBS and 1% penicillin/streptomycin. The cells were maintained at 37°C under 5% CO_2_ in humidified air.

### Flow cytometric analysis

The binding ability of ON-CAG was studied by flow cytometry. Since it was reported that AS1411 could be taken up by SKOV3 cells (human ovarian cancer cells) with high targeting and high affinity (Li et al., 2017), we chose SKOV3 cells as the control for HK-2 cells, and the DNA library (LIB) was used as the control for AS1411. Cells were incubated with 250 nM fluorescein (FAM) labeled LIB, ON, and ON-CAG in 200 μl binding buffer, respectively, at 4°C for 30 min. After incubation, cells were washed twice with washing buffer to remove non-specific aptamers, and cells were resuspended in 200 μl washing buffer before analysis with flow cytometric. The fluorescence intensity was measured using a Backman flow cytometer.

### Cell counting Kit-8

HK-2 cells were seeded in a 96-well plate at a density of 5,000 cells per well and incubated overnight for adherence. Firstly, we incubated HK-2 cells with 20 μmol/L cisplatin. After incubation for 24 h, the solution of cisplatin was removed and cells were treated with different concentrations (1,10,50 μmol/L) of ON or ON-CAG for another 24 h. At the end of the incubation, 10 µl CCK-8 reagent (Beyotime Biotechnology Co., Shanghai, China) was added to each well. The mixture was incubated for 2 h and the absorbance was assessed at 450 nm.

### Enzyme-linked Immunosorbent assay

HK-2 cells were seeded in a 6-well plate at a density of 2 × 10^5^ cells per well and incubated overnight for adherence and cultured with a fresh culture medium containing 20 μmol/L cisplatin for 24 h. The media were then removed, and 2 ml of different concentrations (10 μmol/L) of ON or ON-CAG were added. After another 24 h, the samples were carefully collected to evaluate the protein concentration of IL-6, IL-1β, Kim-1, and IL-18, released from HK-2 cells with commercial ELISA KIT as per the ABCAM manufacturer’s protocol.

### Statistical analysis

Statistical analysis, including a one-way ANOVA test and *t*-test, was carried out using Graph Pad Prism 9 (Graph Pad Software, San Diego, CA, United States). Data were displayed as the mean ± S.E.M. Values of *p* less than 0.05 were considered to be statistical significance.

## Results

### Synthesis of ON-cycloastragenol conjugates

**FIGURE 3:**
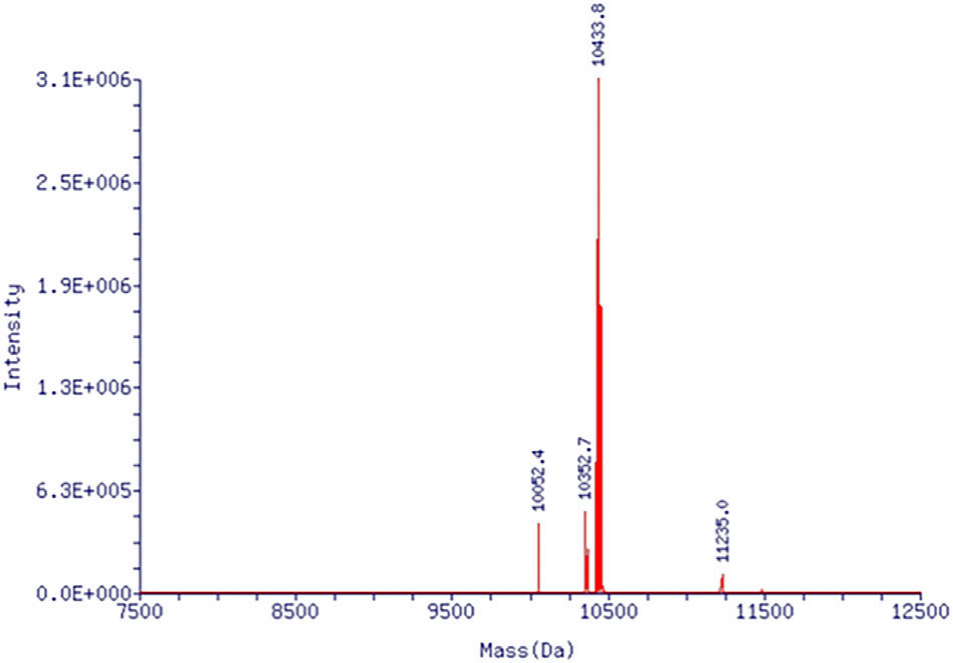
Mass spectrum of ON-CAG conjugates. Mass spectrum showed an exact molecular weight of 10433.8.

From CAG phosphoramidite, fluorescence-labeled ON-CAG (5′-X GGT​GGT​GGT​GGT​TGT​GGT​GGT​GGT​GG (FAM)-3′) was prepared by solid-phase synthesis, in which X presents the CAG unit incorporated from the phosphoramidite. The oligonucleotide was purified by HPLC and identified by mass (Figure 3).

### The binding ability of ON-cycloastragenol with target cells HK-2

**FIGURE 4:**
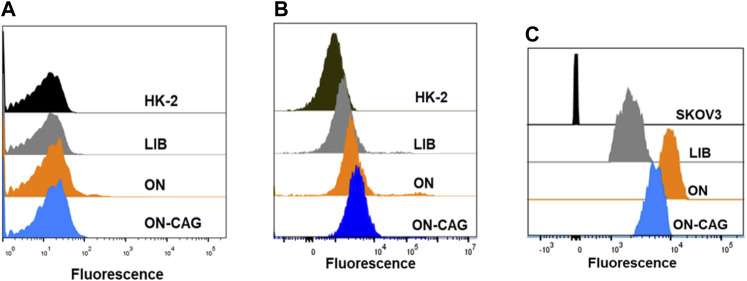
Binding ability of ON-CAG. The cellular uptake of ON-CAG-FAM in normal HK-2 cells **(A)** cisplatin-induced HK-2 cells **(B)** and SKOV3 cells **(C)** monitored by flow cytometry.

To study the binding affinity of ON-CAG to HK-2 cells, the fluorescence intensities of cells were determined by flow cytometry analysis. The proportion of different cells with fluorescence signal enhancement resulting from aptamer binding was compared by analyzing flow cytometric data. Results showed that ON-CAG didn’t bind to normal HK-2 cells or cisplatin-induced HK-2 cells, but significantly bound to SKOV3 cells (Figures 4A–C). The reason for this result might be the low expression of nucleoli on the membrane surface of HK-2 cells and exposure to cisplatin didn’t cause overexpression of nucleoli.

### The effect of ON-cycloastragenol on the cell viability

**FIGURE 5:**
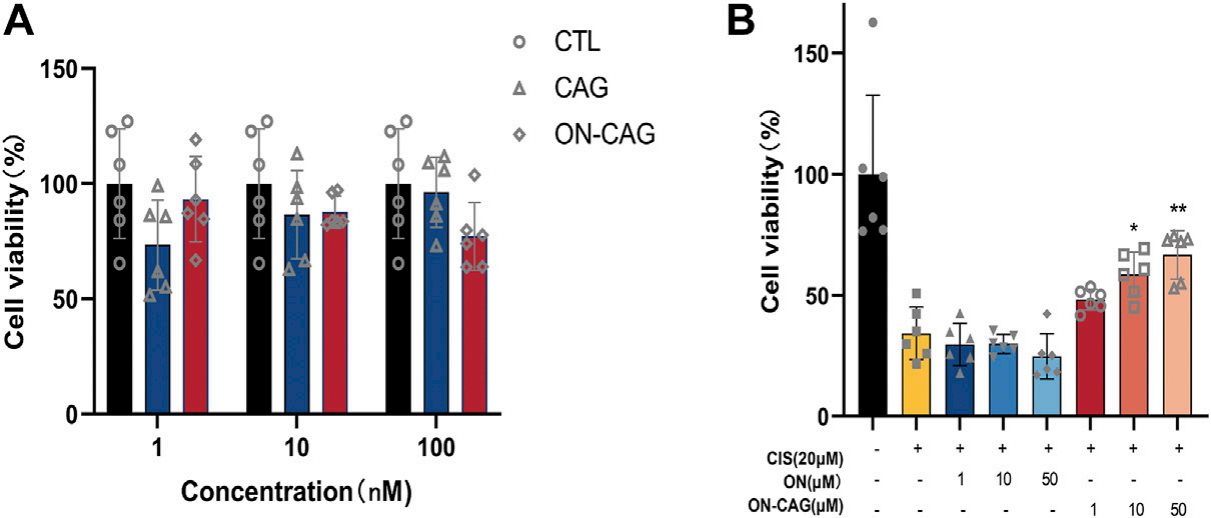
The effect of ON, CAG, and ON-CAG on HK-2 cell viability. **(A)** HK-2 cells were treated with CAG or ON-CAG for 24 h by CCK8 assay, and the concentration of each compound was 1, 10, 100 nmol/L. Data were presented as the mean ± S.E.M. (n = 6). CTL: Control; ON: Oligonucleotides; CAG: Cycloastragenol. **(B)** HK-2 cells were stimulated with 20 μmol/L cisplatin for 24 h and then treated with different concentrations (1,10,50 μmol/L) of ON or ON-CAG for 24 h. Data were presented as the mean ± S.E.M. (*n* = 6). **p < 0.01 compared with the cisplatin group. CTL: Control; CIS: Cisplatin.

The CCK-8 assay was used to evaluate the effect on HK-2 cell proliferation of ON, CAG, and ON-CAG. We incubated HK-2 cells with CAG or ON-CAG separately for 24 h at various concentrations (1,10,100 nmol/L) and measured cell viability. There were no statistically significant differences in growth between the cells exposed to different concentrations of CAG and ON-CAG (Figure 5A, *p* > 0.05). Furthermore, HK-2 cells were stimulated with 20 μmol/L cisplatin for 24 h and then treated with different concentrations (1,10,50 μmol/L) of ON or ON-CAG for 24 h, after which the cell viability was determined. As shown in Figure 5B, cisplatin exhibited considerable cytotoxicity in HK-2 cells compared to the control group (*p* < 0.01). There were no statistically significant differences in growth between the cells exposed to cisplatin and different concentrations of ON (*p* > 0.05). Moreover, the cell viability of HK-2 cells treated with 10 μM and 50 μM ON-CAG was significantly higher than those in the ON group at the same concentration (*p* < 0.01). Thereby, ON-CAG and ON did not affect HK-2 cell proliferation, and ON-CAG could significantly ameliorate cisplatin-induced HK-2 cell cytotoxicity.

### The effect of ON-cycloastragenol on the expression level of Kim-1 and IL-18 protein

**FIGURE 6:**
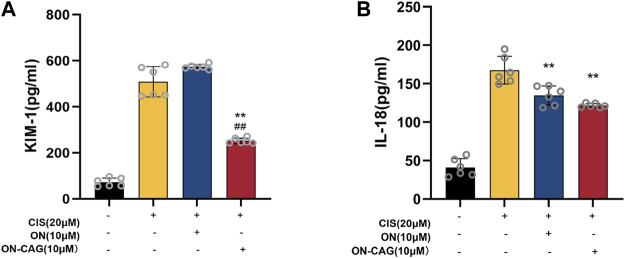
The effect of ON-CAG on cisplatin-induced KIM-1 **(A)** and IL-18 **(B)** expression in HK-2 cells. HK-2 cells were stimulated with 20 μmol/L cisplatin for 24 h and then treated with 10 μmol/L ON or ON-CAG for 24 h. Data were presented as the mean ± S.E.M. **(A)**, *n* = 3; **(B)**, *n* = 6. **p < 0.01 compared with the cisplatin group. ^##^ *p* < 0.01 compared with the ON group.

Both kidney injury molecule-1 (KIM-1) and IL-18 have been proven as valuable biomarkers of renal tubular injury. To explore the possible protective effects of ON-CAG against cisplatin-induced nephrotoxicity, the expression level of KIM-1 protein in each group of HK-2 cells was investigated by ELISA Kit. Cisplatin stimulation increased the expression of KIM-1 protein in HK-2 cells, whereas treatment with 10 μM ON-CAG significantly reduced the upregulation of KIM-1 protein expression induced by cisplatin (Figure 6A, *p* < 0.01). As shown in Figure 6B, the protein concentration of IL-18 released from HK-2 cells in 10 μM ON-CAG was lower than in the cisplatin group (*p* < 0.01). Collectively, our results demonstrated that ON-CAG could effectively protect against cisplatin-induced nephrocyte injury.

### The effect of ON-cycloastragenol on the expression level of IL-6 and IL-1β protein

**FIGURE 7:**
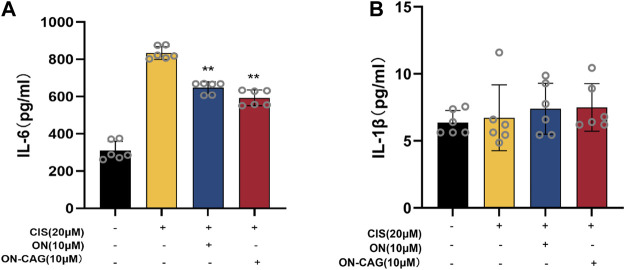
The effect of ON-CAG on cisplatin-induced IL-6 **(A)** and IL-1β **(B)** expression in HK-2 cells. HK-2 cells were stimulated with 20 μmol/L cisplatin for 24 h and then treated with 10 μmol/L ON or ON-CAG for 24 h. Data were presented as the mean ± S.E.M. (*n* = 6).**p < 0.01 compared with the cisplatin group.

The expression level of IL-6 and IL-1β protein in each group of HK-2 cells was investigated by ELISA assay. As can be seen from Figure 7A, the cisplatin group reported significantly more IL-6 protein than the control group (*p* < 0.01), which suggested that cisplatin-induced inflammation in HK-2 cells. Compared with the cisplatin group, the expression level of IL-6 protein in the ON-CAG group was significantly lower (*p* < 0.01). While there were no statistically significant differences in IL-1β protein between the cells exposed to cisplatin, ON, or ON-CAG (Figure 7B, *p* > 0.05). The results indicated that ON-CAG could attenuate cisplatin-induced renal inflammatory response.

## Discussion

Oligonucleotide has attracted considerable attention as a widely used drug carrier in recent years. With the advantages of high targeting, high affinity, and low immunogenicity, AS1411 is considered a remarkable and promising targeted drug delivery and has a good application prospect in the biomedical field. It recognizes nucleolin protein specifically, which predominantly exists in the nucleolus and is also present in cytoplasm and on the cell surface. Nucleolus is essential for the initiation and activation of TGF-β, epidermal growth factor (EGF)-induced multiple pathways (Yazdian-Robati et al., 2020). Furthermore, AS1411 has been tested in over 100 patients with solid tumors or acute myeloid leukemia in Phase I clinical trial and Phase II clinical trial. It turned out that this aptamer showed good overall tolerability and no evidence of severe side effects was observed (Bates et al., 2009; Rosenberg et al., 2014). Its excellent safety profile and targeting allow it to be utilized in a variety of medical fields. Herein, we designed AS1411 as a vector for the targeted delivery of CAG into cells to increase the intracellular concentration and improve the efficacy.

ON-CAG conjugates were successfully synthesized by oligonucleotide synthesis technology, which improve water solubility substantially. However, the results of binding ability are not ideal. ON-CAG appeared better binding force on tumor cells than normal HK-2 cells or cisplatin-induced HK-2 cells. It may be associated with high expression of nucleolin protein in tumor cells. We need to synthesize more ON-CAG and carry out pharmacokinetic studies to reveal its absorption, distribution, metabolism, and elimination inside living organisms. To assess the cytotoxicity of the obtained ON-CAG, a cell viability study based on a CCK-8 reagent assay was performed in HK-2 cells. Our *in vitro* study demonstrated that various doses (1,10,100 nmol/L) of ON-CAG had no cytotoxic effects, and we found no statistically significant differences in growth between the cells exposed to different concentrations of CAG and ON-CAG. Therefore, AS1411 can be used as a stable biosafety carrier for drug delivery.

Studies reported that KIM-1 messenger RNA and protein are increased dramatically in the postischemic kidney and KIM-1 is identified as an *in vitro* biomarker for the evaluation of cisplatin-induced nephrotoxicity (Sohn et al., 2013; Song et al., 2019). IL-18, a kind of pro-inflammatory factor, is expressed in the proximal renal tubule when AKI occurs. It also has been proven as a rapid and affordable test index for the early detection of AKI (Lin et al., 2015; Nozaki et al., 2017). In this study, we measured protein levels of KIM-1 and IL-18 in HK-2 cells using ELISA assay and found that cisplatin increased the expression of KIM-1 and IL-18 protein, while ON-CAG significantly decreased the expression of these proteins. Therefore, our results suggested that ON-CAG possessed renoprotective effects for cisplatin-mediated toxicity.

After entering renal tubular epithelial cells, cisplatin generates a large number of reactive oxygen species, induces an inflammatory response, and causes apoptosis and necrosis of renal tubular cells (Ozkok et al., 2016; Zhu et al., 2020). In agreement with previous reports (Jin et al., 2015; Zhao and Wen, 2018), our study demonstrated that cisplatin exposure could cause HK-2 cell damage. Treatment with ON-CAG resulted in a significant improvement in cell viability and downregulation of IL-6 protein in cisplatin-treated HK-2 cells. These data suggested that ON-CAG could alleviate renal tubular damage and inflammatory response in cisplatin-induced AKI HK-2 cells.

In conclusion, we have designed the solid phase module for CAG and synthesized the phosphoramidite, from which ON-CAG conjugates were prepared efficiently. Our *in vitro* studies proved that the ON-CAG conjugates showed a great renoprotective effect similar to free CAG. This study provides an efficient approach to incorporating TCM monomer CAG into oligonucleotide and significantly improving its water solubility, which may facilitate the discovery of new therapeutics based on TCM.

## Data Availability

The original contributions presented in the study are included in the article/Supplementary Material, further inquiries can be directed to the corresponding authors.
